# Effects of quercetin on the proliferation of breast cancer cells and expression of survivin *in vitro*

**DOI:** 10.3892/etm.2013.1285

**Published:** 2013-09-03

**Authors:** XIAO-HUI DENG, HAI-YAN SONG, YING-FENG ZHOU, GUO-YAN YUAN, FENG-JIN ZHENG

**Affiliations:** 1 Department of Human Anatomy, Xinxiang Medical University, Xinxiang, Henan 453003;; 2 First Affiliated Hospital of Xinxiang Medical University, Weihui, Henan 453000;; 3 West China School of Preclinical & Forensic Medicine, Chengdu, Sichuan 610041, P.R. China

**Keywords:** proliferation, cell cycle, survivin, quercetin, breast cancer

## Abstract

Quercetin is a hydrophobic agent with potential anticancer activity. The aim of the present study was to observe the effects of quercetin on the proliferation of the breast cancer cell line MCF-7 and the gene expression of survivin. The molecular mechanism underlying the antiproliferative effect of quercetin was also investigated. MCF-7 breast cancer cells were treated with various concentrations of quercetin. The inhibitory effect of quercetin on proliferation was detected using the 3-(4,5-dimethylthiazol-2-yl)-2,5-diphenyltetrazolium bromide (MTT) method and the inhibition rate was calculated. Cellular apoptosis was detected by immunocytochemistry and survivin mRNA expression levels were observed using reverse transcription-polymerase chain reaction (RT-PCR). Western blot analysis was used to analyze changes in the expression levels of survivin protein. Quercetin induced the apoptosis of MCF-7 cells and inhibited the proliferation of the MCF-7 breast cancer cells in a time- and concentration-dependent manner. The mRNA and protein expression levels of survivin were reduced as the concentration of quercetin increased. Quercetin inhibited the growth of MCF-7 cells and promoted apoptosis by inducing G0/ G1 phase arrest. It also regulated the expression of survivin mRNA in MCF-7 cells, which may be the mechanism underlying its antitumor effect.

## Introduction

Breast cancer is one of the most common malignancies and it has a serious impact on female health. Morbidity and mortality are high in female malignant tumors (1). According to the World Health Organization, ~1.3 million women are diagnosed with breast cancer each year (2). The incidence rate of breast cancer ranks first among all malignant tumors in women and breast cancer mortality is only lower than that of lung cancer (3,4).

Despite the development of surgical techniques and meticulously designed chemotherapy regimens, relapse remains almost inevitable in patients with advanced cases of the disease. Although there are many chemical therapeutic drugs for the treatment of breast cancer that are able to kill or inhibit the growth of tumors, they usually are associated with a number of side-effects. Therefore, the exploration and development of novel antitumor chemotherapeutics is critical to the improvement of integrated treatment plans.

Quercetin is a polyphenolic compound widely distributed in a number of fruits, vegetables and plants. Previous studies have indicated that quercetin has promising applications in cancer therapy (5–7). It has been reported that quercetin is capable of inhibiting the growth of cancer cells through the induction of apoptosis in a variety of cancer cell lines. Previous studies have demonstrated that quercetin is able to inhibit the proliferation of gastric, esophageal and ovarian cancers (8–10). However, the effects of quercetin on breast cancer have rarely been investigated. Therefore, the aim of this study was to investigate the anticancer effect and mechanism of action of quercetin on breast cancer.

## Materials and methods

### 

#### Materials

The MCF-7 human breast cancer cell line was provided by the Tumor Cell Library of the Chinese Academy of Medical Sciences (Beijing, China). Quercetin (Sigma, St. Louis, MO, USA) was suspended in dimethylsulfoxide (DMSO) and stored at −20°C. 3-(4,5-Dimethylthiazol-2-yl)-2,5-diphenyltetrazolium bromide (MTT) and DMSO were purchased from Sigma. Dulbecco’s modified Eagle’s medium (DMEM), fetal calf serum (FCS) and TRIzol were bought from Invitrogen Life Technologies (Carlsbad, CA, USA). Hoechst 33258 was provided by Biyuntian Biotechnology (Haimen, China).

### Methods

#### Cell culture

MCF-7 cells were cultured in 10% DMEM (containing 10% FCS) at 37°C in 5% CO_2_ and under saturated humidity conditions. The medium was replaced every two or three days.

#### Cell viability and proliferation

MCF-7 cells were harvested during the logarithmic growth phase and digested with trypsin. To study the effects of quercetin on cell proliferation and viability, MCF-7 cells (5×10^3^/well) were plated in 96-well plates and incubated in DMEM supplemented with 10% FCS. After 24 h, cells were washed once with medium and treated with 0, 2.5, 5, 10, 20 and 40 mg/ml quercetin in the medium. Control wells contained MCF-7 cells without quercetin and blank wells contained only culture medium. Cell proliferation and cell viability were determined after 24 or 48 h of treatment by incubation in DMEM, supplemented with 10% FCS containing 20 *μ*l MTT (5 mg/ml), for 4 h. The culture solution was swilled, 150 *μ*l DMSO was added to each well and the solution was subsequently shaken to completely dissolve the blue-purple precipitate obtained from MTT. A microplate reader (OPTImax; Molecular Dynamics, Sunnyvale, CA, USA) was used to test the absorbance (A) of each well at 540 nm and average values were obtained. Experiments were repeated ≥3 times and data are presented as the mean ± SD.

#### Analysis of cell apoptosis

In order to investigate the apoptosis-inducing effect of quercetin, morphological analysis was carried out following staining with Hoechst 33258. Apoptosis of MCF-7 cells was detected by flow cytometry (Axiovert 200; Carl Zeiss SMT GmbH, Oberkochen, Germany). The cells were collected following treatment with 0, 2.5, 5, 10, 20 and 40 mg/ml quercetin. Subsequently, the cells (1×10^6^) were centrifuged at 700 × g for 5 min and the supernatants were discarded. The cells were washed twice with phosphate-buffered saline (PBS), 70% alcohol was added and the mixture was centrifuged after 30 min at 700 × g for 5 min. After washing with PBS twice, 1 ml propidium iodide (PI) was added, and the cells were incubated for 30 min in the dark at 25°C. Cell apoptosis was then detected using a flow cytometer (FCM: BD FACSCalibur; BD Biosciences, San Jose, CA, USA).

#### Measurement of survivin mRNA expression

The expression of survivin mRNA was detected by reverse transcription-polymerase chain reaction (RT-PCR). The survivin and H-GAPDH primers were designed using Primer 5.0 (Premier Biosoft International, Palo Alto, CA, USA), with H-GAPDH as the internal reference. Total RNA was extracted and purified using the TRIzol kit and subsequently synthesized by two-step RT-PCR. The PCR parameters were as follows: 33 cycles of denaturation at 95°C for 30 sec, annealing at 54°C for 45 sec, and extension at 72°C for 1 min.

#### Detection of survivin protein expression

Survivin protein was detected by western blot analysis. MCF-7 cells were treated with different concentrations of quercetin for 24 h and subsequently cultured for 6 h following a change of medium. Different groups of cells were collected in order to extract and quantify the protein. The protein was separated by sodium dodecyl sulfate-polyacrylamide gel electrophoresis (SDS-PAGE) and then transferred to a blocked nitrocellulose (NC) membrane for 1 h with 5% skimmed dry milk. The membrane was incubated with rabbit anti-human survivin antibody, diluted (1:1,000) in blocking buffer overnight at 4°C and then washed with Tris-buffered saline Tween (TBST, 3×10 min). Subsequently, the membrane was incubated with TBST for 3×10 min. An ECL Western Blotting kit (Suzhou JiShi biological technology company, Suzhou, China) was used for detection.

#### Statistical analysis

Data are presented as the mean ± SD. Statistical analysis was performed with a one-way analysis of variance (ANOVA) using SPSS software (SPSS, Inc., Chicago, IL, USA). P<0.05 was considered to indicate a statistically significant difference.

## Results

### 

#### Inhibitory effect of quercetin on the proliferation of MCF-7 cells

**Figure 1.**
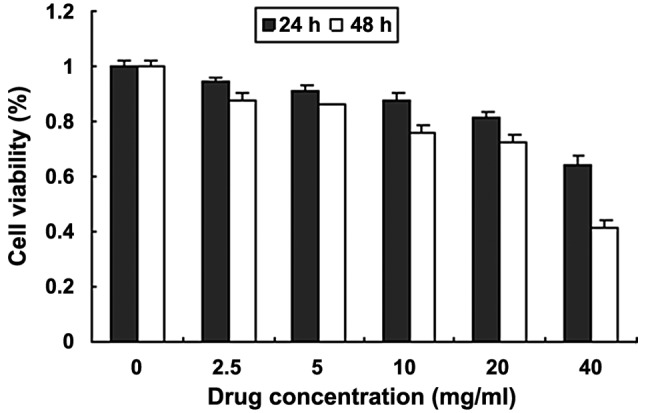
Inhibitory effect of quercetin on the proliferation of MCF-7 cells.

Fig. 1 demonstrates the inhibitory effect of quercetin on the proliferation of MCF-7 cells. The results showed that cell growth activity was reduced and proliferation was inhibited. The viability of the cells was reduced when the incubation time was extended for the same dose. The minimum cell activity was observed following treatment with 40 mg/ml quercetin for 48 h. The highest inhibition rate was 58.72% and the rate of inhibition was concentration- and time-dependent.

#### Effect of quercetin on apoptosis of MCF-7 cells

**Figure 2.**
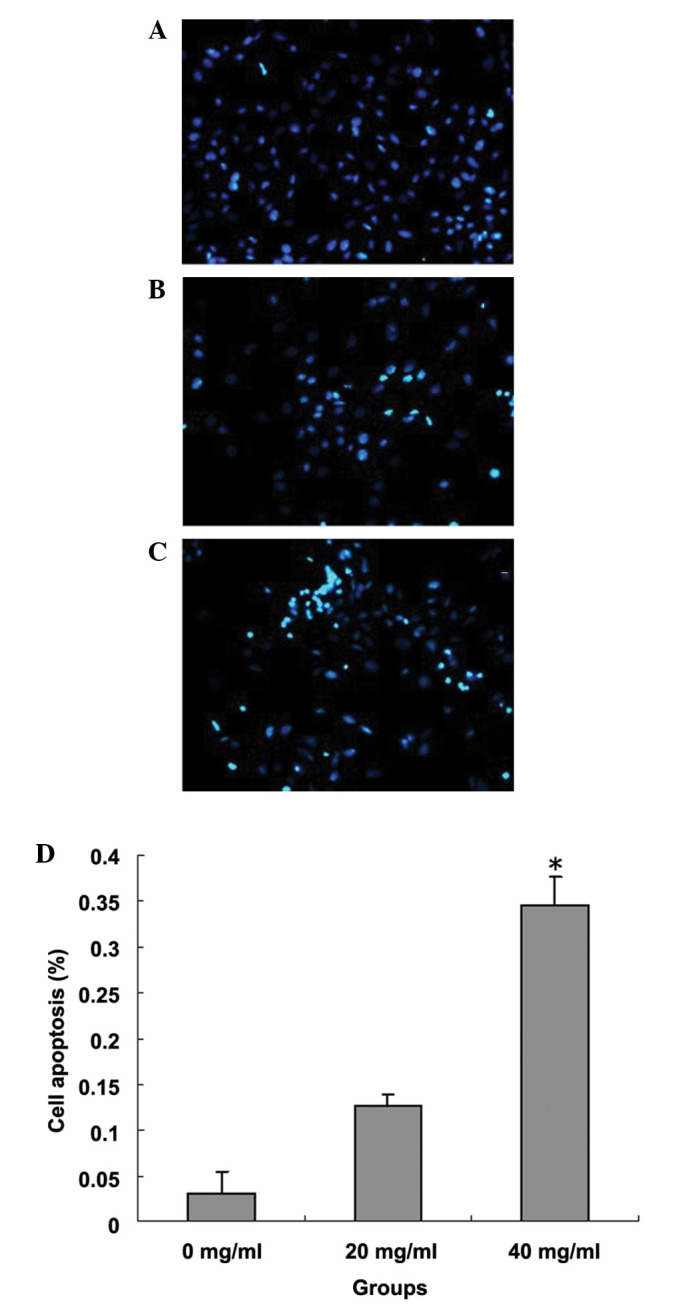
Results of Hoechst 33258 staining (magnififcation, ×200). (A) Normal group (0 mg/ml quercetin); (B) 20 mg/ml quercetin group; and (C) 40 mg/ml quercetin group. (D) With the increase of drug concentration cell apoptosis rate also increases

Table I shows that quercetin induced the apoptosis of breast cancer cells in a concentration-dependent manner. The number of cells (40.24 and 59.71%) in the G0/G1 phase significantly increased in the quercetin-treated cells compared with that of the control group. The apoptosis rate of the quercetin 40 mg/ml group was 37.81%, which was significantly higher than the apoptosis rates in the low concentration (20 mg/ml) and control groups. Fig. 2 shows that when cells were stained with Hoechst 33258 and observed under a fluorescence microscope, increased levels of nuclear fragmentation and apoptotic bodies (bright-blue) were detected in the cells treated with quercetin.

#### Expression of survivin mRNA in MCF-7 cells

**Figure 3.**
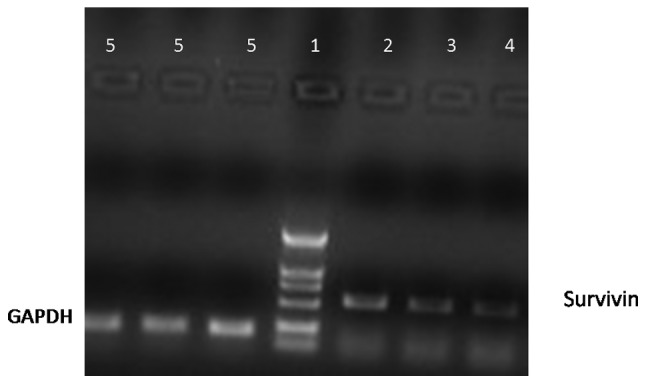
Expression of survivin mRNA in MCF-7 cells. Lane 1, marker; lane 2, 0 mg/ml quercetin; lane 3, 20 mg/ml quercetin; lane 4, 40 mg/ml quercetin; lane 5, GAPDH.

Fig. 3 shows that the survivin mRNA levels were reduced when the concentration of quercetin increased.

#### Expression of survivin protein

**Figure 4.**
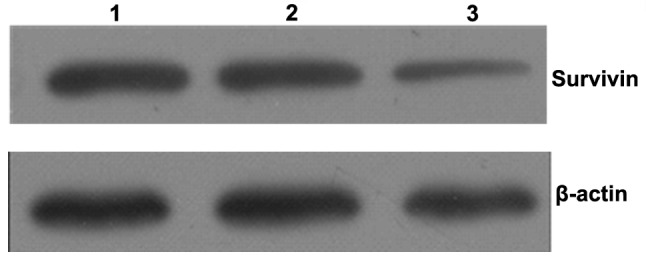
Expression of survivin protein in MCF-7 cells. Lane 1, normal group (0 mg/ml quercetin); lane 2, 20 mg/ml quercetin; lane 3, 40 mg/ml quercetin.

Survivin expression levels were examined by western blot analysis of MCF-7 cells following quercetin treatment for 48 h. As shown in Fig. 4, reductions in survivin protein levels were observed following quercetin treatment. This corresponds with the changes observed in the levels of survivin mRNA.

## Discussion

Due to the high incidence and high fatality rates of breast cancer in recent years, the development of an effective method of treatment is urgently required. Bio-flavonoids extracted from fruits and vegetables have a variety of biological properties, including antioxidant, antibacterial, anti-inflammatory, antiviral, anticancer and cancer preventing activities (11,12). It has been demonstrated that the flavonoid quercetin inhibits the proliferation of colon, pancreas, stomach, bladder and ovarian cancers, as well as the proliferation of other types of tumor cells, and induces tumor cell apoptosis (13–15).

The antitumor effect of quercetin on breast cancer cells *in vitro* was detected using an MTT assay and flow cytometry. It was observed that quercetin treatment led to cells remaining in the G0/G1 phase and significantly reduced the proportion of cells in the G2 phase. This effectively inhibited breast cancer cell proliferation and prompted cells apoptosis. The inhibitory effect of quercetin on cancer cell proliferation increased as the drug concentration and length of action time increased. Quercetin caused a concentration- and time-dependent reduction in the viability of MCF-7 human breast cancer cells.

Survivin is a member of a protein family responsible for apoptosis inhibition (inhibitor of apoptosis protein, IAP). It has a simple and unique molecular structure, and has been identified to be the IAP with the strongest ability to inhibit apoptosis (16,17). Survivin exhibits high expression levels in a number of tumors and is not expressed at all in normal adult tissues (with the exception of placenta and thymus tissue). Ryan *et al* (18) demonstrated that the degree of survivin expression correlated with tumor progression, the degree of malignancy and pathology classification, and that survivin may be used in relation to breast cancer diagnosis and in indicating prognosis.

The present study investigated the effects of quercetin as an inhibitor of the proliferation and apoptosis of MCF-7 breast cancer cells. Furthermore, the potential antitumor effect of quercetin in breast cancer treatment was evaluated by measuring the expression level of survivin mRNA. RT-PCR demonstrated that the expression level of survivin mRNA was reduced in MCF-7 human breast cancer cells following treatment with quercetin. Western blot analysis revealed that the level of survivin protein also decreased. The survivin mRNA and protein levels were negatively correlated with quercetin concentration. The results indicate that quercetin may be capable of improving the sensitivity of breast cancer cells to chemotherapy by decreasing the expression level of survivin mRNA in breast cancer cells. Furthermore, during the breast cancer treatment process, the detection of survivin expression levels in tumor tissue may be used to determine the effectiveness of treatment. Survivin expression may not only provide a basis for clinical diagnosis of breast cancer, but also may be used as an indicator to estimate the prognosis of breast cancer.

## Figures and Tables

**Table I.** Effect of quercetin on the cell cycle and apoptosis of MCF-7 cells (mean ± SD).

Group	Cell cycle (%)			Apoptosis rate (%)
	G0/G1	S	G2/M	
0 mg/ml	37.81±0.51	31.24±0.14	30.95±0.34	1.03±0.14
20 mg/ml	40.24±0.33	27.15±0.05	32.61±0.24	7.31±0.21
40 mg/ml	59.71±0.16	16.27±0.21	24.02±0.29	27.11±0.27^a,b^

a P<0.05 and

b P<0.01 vs. the control group.
